# Prevalence of Meibomian Gland Dysfunction in Patients with Keratoconus in an Optometry Clinic in the Palestinian Authority

**DOI:** 10.3390/biomedicines14010134

**Published:** 2026-01-09

**Authors:** Reut Ifrah, Taqwa Darwish

**Affiliations:** Department of Optometry and Vision Science, Jerusalem Multidisciplinary College, Jerusalem 9101001, Israel; takwada@edu.jmc.ac.il

**Keywords:** keratoconus, meibomian gland dysfunction, palestinian authority, meibomian gland loss

## Abstract

**Background/Objectives:** Meibomian gland dysfunction (MGD) has been shown to be more prevalent in patients with keratoconus (KC) in Turkey, Egypt, and Israel but has not been examined in the Palestinian Authority (PA). Therefore, this study compared the prevalence and clinical features of MGD in patients with keratoconus versus healthy controls seen in an optometry clinic in the PA. **Methods:** Patients with KC and healthy controls who were non-contact lens wearers were recruited. Habitual visual acuity (VA), tear break-up time (TBUT), meibography, meibomian gland (MG) expressibility (MES) and quality score (MQS), and Schirmer test were evaluated. MGD was defined by an Ocular Surface Disease Index (OSDI) Questionnaire score ≥ 13, TBUT < 10 s, and MG loss > Grade 1. Outcomes were compared using Mann–Whitney U tests, Chi-Square tests and Spearman correlation. **Results:** The study included 33 eyes of 17 KC (mean age: 29.2 ± 7.7, range:19–50) and 27 right eyes of 27 control participants (mean age: 34.2 ± 11.7, range:18–56). MGD was prevalent in 67% of KC and 30% of control participants. VA was significantly worse (0.8 + 0.2 vs. 1.0 + 0.0, *p* < 0.001), with significantly greater MG loss in the lower eyelids (*p* = 0.002), and shorter TBUT (4.1 ± 1.5 s vs. 5.7 ± 1.7 s, *p* < 0.001) in the KC group. No significant differences were found in symptoms, MES, MQS, MG loss in the upper eyelids, or Schirmer test. **Conclusions:** KC patients exhibited a significantly higher prevalence and severity of MGD signs compared with controls. These findings highlight the importance of comprehensive ocular surface evaluation and management in this population.

## 1. Introduction

Keratoconus (KC) and Meibomian Gland Dysfunction (MGD) are prevalent ocular conditions that can substantially affect visual function and overall quality of life [1,2].

KC is the most common ectatic corneal disorder, typically affecting both eyes asymmetrically [3,4]. KC is characterized by progressive corneal thinning and conical protrusion that result in irregular astigmatism and vision impairment [4,5,6]. The reported prevalence of KC varies widely, ranging from 0.0003% in Russia to 2.3% in Maharashtra, India [7]. In Nablus (Palestinian Authority) and Jerusalem, prevalence rates of 1.5% and 2.3% have been reported, respectively [7,8], while in the broader Middle East, prevalence has generally been shown to exceed 2.0% [8,9,10]. The exact etiology of KC remains unclear, although genetic, environmental, and biomechanical factors are believed to contribute to its development [11,12].

The meibomian glands (MGs) play a critical role in maintaining ocular surface homeostasis by secreting lipids that form the outer layer of the tear film, reducing tear evaporation and stabilizing the tear film during blinking [13,14]. Adequate patency of these glands is essential for preserving a smooth optical surface and protecting the ocular surface from desiccation and inflammation [15,16].

MGD, a major contributor to dry eye (DE) disease, is a chronic abnormality of the MGs characterized by duct obstruction and/or changes in glandular secretion, leading to reduced lipid production, altered tear film, and ocular surface inflammation [17]. Reported prevalence of MGD varies widely, ranging from 3.5% to 69.3%, depending on study design, population, environmental factors, and diagnostic criteria [18,19] with high prevalence observed in the Middle East [20]. In a study conducted in Egypt, the prevalence of MGD was reported to be 18.5% [21] whereas in Iran it was found to be 26.3% [22]. Notably, even higher prevalence has been reported in Saudi Arabia, with a prevalence of 76.1% [23].

Although KC and MGD are distinct ocular conditions, emerging evidence suggests a potential association between them [6,24,25,26]. MGD can lead to persistent eye rubbing, which may contribute to the pathogenesis of KC [6]. Further, several studies have reported a higher prevalence of dry eye and MGD in patients with KC compared to the general population, highlighting a possible interplay that may influence disease progression and management [6,24,26,27,28].

The coexistence of MGD in KC patients may exacerbate ocular surface inflammation, destabilize the tear film, and compromise corneal integrity, resulting in increased discomfort and reduced visual acuity [6,25].

Despite these potential interactions, the literature addressing the coexistence of KC and MGD remains limited, with most studies examining the conditions in isolation. To the best of our knowledge, no study has specifically investigated the prevalence of MGD in a clinical population seen in an optometry clinic in the Palestinian Authority. Therefore, this study aimed to compare the prevalence of MGD among KC patients and non-KC patients in the Palestinian Authority.

## 2. Materials and Methods

This retrospective study conformed to the ethical principles of the Declaration of Helsinki and was approved by the internal ethics committee of Jerusalem Multidisciplinary College (JMC, formerly Hadassah Academic College, approval number 680), Israel.

### 2.1. Participants

The study included files of patients with KC who had been previously diagnosed by an ophthalmologist, as well as a control group of healthy patients without KC. Only files of non-contact lens wearers were reviewed. Files were excluded if they documented a history of ongoing ocular surface disease; allergic, atopic, or vernal keratoconjunctivitis; use of systemic medications associated with dry eye or MGD (e.g., antihistamines, hormones) or pregnancy.

### 2.2. Procedures

The files of patients seen at Ibn AL_Haitham Optics, Ramallah, Palestinian Authority were included in this study. All patients were previously examined in a designated examination room, by a certified optometrist (TD). After reviewing the medical and ocular history on the chart to verify eligibility for inclusion, the habitually corrected distance visual acuity (Snellen chart), corneal topography (Rexxam, Tokyo, Japan; https://www.rexxam.co.jp/eye-care/products/ret700.html, accessed on 4 January 2026) and slit lamp biomicroscopy (LS/4 from Redsun, Nanjing, China; https://www.redsunoptical.com/LS-4-Ophthalmic-Equipment-China-Biomicroscope-Slit-Lamp-pd6710579.html, accessed on 4 January 2026) findings were extracted from the patient files. Based on the clinical outcomes, KC severity was graded according to the Amsler–Krumeich classification [29,30].

The patient’s Ocular Surface Disease Index (OSDI) score [31] was also extracted from the files. The OSDI provides a quantitative measure of the impact of DE on patients’ quality of life, with higher scores reflecting more severe symptomatology [32].

In addition, the tear film break-up time (TBUT), meibum quality score (MQS), meibum expressibility score (MES), and Schirmer test scores were extracted from the patients’ files.

The TBUT scores in the patient files represent the mean of three measurements obtained by instilling a fluorescein strip into the inferior tear meniscus, after which the interval between a complete blink and the first appearance of tear film disruption was recorded under cobalt blue illumination [33]. A TBUT of less than 10 s was considered abnormal [34].

The MQS indicates the meibum excreted from the eight central glands of the lower tarsus when applying pressure for 10 s. MQS was graded as follows: 0 = clear fluid; 1 = cloudy fluid; 2 = cloudy fluid with particulate matter; and 3 = thick secretion resembling toothpaste [34]. MES was graded on the following scale: 0 = all glands expressible; 1 = three to four glands expressible; 2 = one to two glands expressible; and 3 = no glands expressible [34].

Images of the MGs of both eyelids of both eyes was extracted from the Rexxam device. MG loss was graded subjectively using the 0–4 meiboscale (0 = no loss; 4 = severe loss) [35].

The Schirmer test score on the chart represented Schirmer testing without topical anesthesia which was obtained after placing a paper strip in the mid-lateral lower fornix with the patient’s eyes closed. The length of strip wetting after 5 min was recorded, with a cutoff value of <10 mm considered abnormal [33,34].

Dry eye prevalence was defined based on the TFOS DEWS II report [36]. Participants with an OSDI score ≥ 13 and TBUT < 10 s were considered participants with dry eye. They were further defined as having MGD based on the International Work-shop [37] on MGD if their MG loss was greater than Grade 1.

### 2.3. Statistical Analysis

Using G-Power software (version 3.1.9.7; Heinrich-HeineUniversität Düsseldorf, https://www.psychologie.hhu.de/arbeitsgruppen/allgemeine-psychologie-und-arbeitspsychologie/gpower, accessed on 31 October 2025) [38] when comparing two cohorts of 60 eyes that are not normally distributed with an error probability of 5% (α error 0.05) and an effect size (w) of 0.39, the power (1−β) is 85%. KC is an asymmetric disease [3,4] therefore both eyes of each patient were included [39]. Demographic data were evaluated using descriptive statistics. The means, standard deviations, medians and quartiles of the outcome measures were calculated. The normality of the outcome measures was assessed using the Kolmogorov–Smirnov test. Categorical variables were compared amongst subgroups using Chi-Square tests. Continuous variables were compared amongst subgroups using Mann–Whitney tests as the outcomes were abnormally distributed. Relationships between variables such as the KC degree and MG loss were examined using Spearman correlation due to data sets that were not normally distributed. Statistical analyses were performed using SPSS version 27 (SPSS Inc., Chicago, IL, USA) and R Statistical Software (v4.3.1; R Core Team 2021, R Foundation for Statistical Computing, Vienna, Austria).

## 3. Results

The files of 17 KC patients (both eyes of most were included for a total of 33 eyes), aged 19–50 years, with a mean age of 29 ± 8 years, and 27 control patients (27 right eyes), aged 18–56 years, with a mean age of 34 ± 12 years, were included. Their demographic data is shown in Table 1.

A chi-square test of independence showed no significant difference between the groups in the number of females (X^2(1)^ = 0.17, *p* = 0.68). Likewise, no significant age differences were observed between the cohorts (Mann–Whitney test, *p* = 0.25, Table 1).

Among the KC participants, 17 eyes were classified as stage 1, 13 eyes as stage 2, none as stage 3, and three eyes as stage 4, according to the Amsler–Krumeich classification [29,30]. KC grade was negatively correlated with both TBUT (rho = −0.47, *p* < 0.001) and VA (rho = −0.62, *p* < 0.001). KC grade was positively correlated with MG loss in the lower eyelids (rho = 0.38, *p* = 0.003).

The controls had significantly better visual acuity (*p* < 0.001), lower refractive cylindrical component (*p* < 0.001) and keratometry readings (*p* < 0.001) compared with the KC group. The groups did not differ in their refractive spherical component (*p* = 0.42) or their OSDI scores (*p* = 0.21, Table 2).

Morphologically, the degree of MG loss in the lower eyelids was higher in the KC group compared with the control group (*p* = 0.002, Post Hoc Bonferroni: Degree 0, control > KC, *p* = 0.01), though the MG loss in the upper eyelids was not significantly different (*p* = 0.23, Table 2).

Functionally, TBUT was significantly lower in the KC compared with the controls (*p* < 0.001), whereas MQS and MES did not differ significantly between the groups (*p* = 0.74 and 0.26, respectively, Table 2).

No significant inter-ocular differences were observed in MGD grading or related clinical parameters among KC patients (Table 3).

### Prevalence of Dry Eye and MGD

The prevalence of dry eye was not significantly different (*p* = 0.74) between the KC (85%; 28/33 eyes) and control groups (87%; 22/27 eyes). The prevalence of MGD in the KC (22/33 eyes) was significantly greater than the controls (8/27 eyes) (67% vs. 30%, *p* = 0.004).

**Table 2:** Clinical outcome measures of all participants and of the keratoconus and control groups. The means (±standard deviations), ranges, medians, and first and third quartiles (Q1, Q3) for each outcome measure are detailed.

Outcome Measure			Keratoconus	Control	*p*	All Participants
Distance Visual Acuity (Snellen Decimal)		Mean ± SD	0.83 ± 0.19	1.00 ± 0.00	<0.001	0.91 ± 0.17
		Range	0.30–1.00	1.00–1.00		0.30–1.00
		Median	0.80	1.00		1.00
		(Q1, Q3)	(0.70, 1.00)	(1.00, 1.00)		(0.80, 1.00)
Spherical Refraction (D)		Mean ± SD	−0.94 ± 2.16	−0.21 ± 2.36	0.42	−0.61 ± 2.26
		Range	−9.50–1.50	−5.50–8.00		−9.50–8.00
		Median	−0.25	0.00		0.00
		(Q1, Q3)	(−1.50, 0.50)	(−1.00, 0.50)		(−1.50, 0.50)
Cylindrical Refraction (D)		Mean ± SD	−2.45 ± 1.36	−0.64 ± 0.86	<0.001	−1.63 ± 1.47
		Range	−5.50–0.00	−3.00–0.00		−5.50–0.00
		Median	−2.50	−0.25		−1.50
		(Q1, Q3)	(−3.50, −1.50)	(−1.25, 0.00)		(−2.50, −0.13)
Keratometry (D)		Mean ± SD	46.30 ± 3.18	42.86 ± 1.23	<0.001	44.75 ± 3.02
		Range	41.92–55.75	39.38–44.75		39.38–55.75
		Median	46.13	43.00		44.00
		(Q1, Q3)	(44.51, 47.75)	(42.25, 43.75)		(42.63, 46.54)
OSDI score (0–100)		Mean ± SD	32.52 ± 15.31	26.69 ± 15.71	0.21	28.94 ± 15.64
		Range	10.00–60.10	00.00–62.50		0.00–62.50
		Median	34.10	22.70		24.98
		(Q1, Q3)	(17.71, 43.10)	(16.60, 35.10)		(16.60, 41.60)
TBUT (sec)		Mean ± SD	4.06 ± 1.48	5.67 ± 1.69	<0.001	4.78 ± 1.76
		Range	2.00–7.00	2.00–9.00		2.00–9.00
		Median	4.00	6.00		5.00
		(Q1, Q3)	(3.00, 5.00)	(5.00, 7.00)		(4.00, 6.00)
Schirmer test (mm)		Mean ± SD	18.42 ± 6.92	19.00 ± 8.83	0.64	18.68 ± 7.77
		Range	5.00–35.00	3.00–35.00		3.00–35.00
		Median	17.00	20.00		18.50
		(Q1, Q3)	(15.00, 25.00)	(15.00, 25.00)		(15.00, 25.00)
MG loss (0–4)	Upper eyelid	0	7 (21%)	8 (30%)	0.23	15 (25%)
		1	12 (36%)	13 (48%)		25 (42%)
		2	8 (24%)	4 (15%)		12 (20%)
		3	6 (18%)	1 (3.7%)		7 (12%)
		4	0 (0%)	1 (3.7%)		1 (1.7%)
	Lower eyelid	0	3 (9.1%)	12 (44%)	0.002	15 (25%)
		1	5 (15%)	8 (39%)		13 (22%)
		2	13 (39%)	4 (15%)		17 (28%)
		3	9 (27%)	2 (7.4%)		11 (18%)
		4	3 (9.1%)	1 (3.7%)		4 (6.7%)
MQS (0–3)		0	23 (70%)	21 (78%)	0.74	44 (73%)
		1	7 (21%)	5 (19%)		12 (20%)
		2	3 (9.1%)	1 (3.7%)		4 (6.7%)
		3	0 (0%)	0 (0%)		0 (0%)
MES (0–3)		0	15 (45%)	18 (67%)	0.26	33 (55%)
		1	15 (45%)	7 (26%)		22 (37%)
		2	3 (9.1%)	2 (7.4%)		5 (8.3%)
		3	0 (0%)	0 (0%)		0 (0%)

**Table 3:** Inter-ocular analysis among keratoconus patients. The means (±standard deviations), ranges, medians, and first and third quartiles (Q1, Q3) for each outcome measure are detailed.

Outcome Measure		OD	OS	*p*
KC grade	Mean ± SD	1.69 ± 0.79	1.69 ± 1.01	0.95
	Range	1.00–4.00	1.00–4.00	
	Median	2	1	
	(Q1, Q3)	(1.00, 2.00)	(1.00, 2.00)	
Distance Visual Acuity (Snellen Decimal)	Mean ± SD	0.81 ± 0.19	0.84 ± 0.21	0.65
	Range	0.40–1.00	0.30–1.00	
	Median	0.8	0.9	
	(Q1, Q3)	(0.70, 1.00)	(0.73, 1.00)	
Cylindrical Refraction (CYL)	Mean ± SD	−2.48 ± 1.34	−2.53 ± 1.37	0.94
	Range	−5.00–0.00	−5.50–0.00	
	Median	−2.25	−2.5	
	(Q1, Q3)	(−3.75, −1.56)	(−3.50, −1.63)	
Keratometry (D	Mean ± SD	46.31 ± 3.31	46.52 ± 3.1	0.59
	Range	41.92–55.75	42.08–51.54	
	Median	46.26	46.38	
	(Q1, Q3)	(44.63, 47.02)	(43.6, 48.38)	
TBUT (sec)	Mean ± SD	4.00 ± 1.63	4.00 ± 1.32	>0.99
	Range	2.00–7.00	2.00–7.00	
	Median	4.5	4.9	
	(Q1, Q3)	(2.00, 5.00)	(3.00, 4.75)	
MG LOSS Upper eyelid	Mean ± SD	1.44 ± 1.03	1.38 ± 1.09	0.74
	Range	0.00–3.00	0.00–3.00	
	Median	1	1	
	(Q1, Q3)	(1.00, 2.00)	(0.25, 2.00)	
MG LOSS Lower eyelid	Mean ± SD	2.00 ± 1.03	2.25 ± 1.18	0.21
	Range	0.00–4.00	0.00–4.00	
	Median	2	2.5	
	(Q1, Q3)	(2.00, 2.75)	(1.00, 3.00)	
MQS	Mean ± SD	0.44 ± 0.73	0.38 ± 0.62	0.32
	Range	0.00–2.00	0.00–2.00	
	Median	0	0	
	(Q1, Q3)	(0.00, 1.00)	(0.00, 1.00)	
MES	Mean ± SD	0.63 ± 0.62	0.69 ± 0.70	0.32
	Range	0.00–2.00	0.00–2.00	
	Median	1	1	
	(Q1, Q3)	(0.00, 1.00)	(0.00, 1.00)	

## 4. Discussion

The present study retrospectively compared the prevalence and clinical features of MGD in KC patients vs. healthy controls seen in an optometric clinic in the Palestinian Authority. MGD was significantly more prevalent and severe among KC patients, with greater MG loss in the lower eyelids and shorter TBUT.

These results are consistent with previous reports suggesting an association between KC and ocular surface abnormalities [24,26]. Martínez-Pérez et al. [24] examined 120 KC patients (mean age: 35 ± 11) and 87 controls (mean age: 37 ± 14) in Spain, showing that MGD was more frequent in KC group (49% vs. 29%). MGD was more common among 50 KC participants from Israel (40%) compared with 72 age-matched healthy controls (6%; *p* < 0.001) [27]. Greater MG loss was also reported among 300 KC patients (mean age: 19 ± 12) compared with 100 healthy controls (mean age: 21 ± 14) in Egypt [26]. Furthermore, alterations in MG morphology and function were observed in 100 KC patients (mean age: 23 ± 6) compared with 100 healthy controls (mean age: 24 ± 7) in Turkey, with significantly higher meiboscores, partial gland loss, gland thickening, and worse tear film parameters [40]. The present study extends these findings by providing outcomes from the Palestinian Authority which is in a geographical region characterized by a high prevalence of both KC [8,9,10] and MGD [20,21,22].

An important observation is that the KC participants in this study were non-contact lens wearers, a characteristic often associated with earlier stages of KC [41]. This is consistent with the relatively low Amsler–Krumeich grading observed in the cohort. The finding that the prevalence of MGD was 2.23 times higher in this early-to-middle stage KC group compared with non-KC participants suggests an association between the two conditions; however, no conclusions regarding causality or disease sequence can be drawn from this cross-sectional analysis.

The high prevalence of MGD observed in the KC cohort in the Palestinian Authority may be influenced by region-specific environmental and demographic factors. The region is characterized by high ultraviolet exposure [42] arid conditions [43] and fluctuating humidity [44] all of which can exacerbate ocular surface stress and contribute to MGD and tear film instability [45,46]. Furthermore, genetic or ethnic predispositions within the local population may modulate susceptibility to both KC and MGD [20,47].

Despite the objective evidence of MG loss and reduced TBUT, OSDI scores did not differ significantly between the groups. This apparent discordance between signs and symptoms has also been highlighted in other studies of dry eye disease and MGD [48,49,50] suggesting that subclinical ocular surface changes in KC may precede the onset of symptomatic complaints. Further, the OSDI score does not distinguish between evaporative dry eye and aqueous deficiency [51] which may partially account for the lack of correlation between clinical findings and patient-reported symptoms in our cohort.

Previous studies have reported an increased prevalence of dry eye in KC patients [28,41,52]. In the present study, the absence of significant differences in Schirmer test results suggests that aqueous tear deficiency is unlikely to be a major contributor. In contrast, reduced tear film stability and increased MG loss in the KC group support a predominantly evaporative form of dry eye, likely driven by MGD.

MG loss was significantly greater in the lower eyelids of KC patients but not in the upper eyelids. This asymmetry has been described in previous studies [48,49,53,54,55] possibly reflecting differences in lid anatomy [56] blink dynamics [57] or mechanical trauma related to habitual eye rubbing [58].

Chronic mechanical eye rubbing, a characteristic feature of MGD [6] is a recognized risk factor for KC progression [59]. This repetitive microtrauma may directly contribute to corneal deformation and cone formation [6,27]. In addition, MGD-associated ocular surface inflammation and elevated inflammatory mediators further promote corneal pathology [27,60]. Together, these factors create to a vicious cycle, in which ocular surface irritation drives more rubbing, thereby exacerbating KC development and progression [27,61].

Importantly, no significant inter-ocular differences were observed in MG loss or related clinical parameters among KC patients, suggesting that the increased prevalence of MGD reflects a bilateral ocular surface condition rather than being driven by asymmetry in KC severity between eyes.

Despite contributing original clinical data from an understudied geographic region, this study has several limitations that should be acknowledged. The relatively small sample size and the single-center design may limit the generalizability of the findings to broader populations. In addition, eye selection bias cannot be excluded, as both eyes of most KC patients were included while only one eye per control participant was analyzed, potentially inflating within-subject correlations. Furthermore, although validated grading systems were used, the subjective assessment of MG morphology may introduce inter- and intra-observer variability. Finally, the cross-sectional nature of the study precludes conclusions regarding causality or temporal relationships between MGD and KC.

## 5. Conclusions

In conclusion, KC patients exhibited a higher prevalence and severity of MGD compared with controls, particularly in the lower eyelids, and demonstrated reduced tear film stability. These findings underscore the importance of comprehensive ocular surface assessment in KC management. Clinicians should be aware of the coexistence of MGD in KC patients, as addressing ocular surface disease may improve comfort, reduce eye rubbing, and potentially mitigate disease progression. Future studies with larger, longitudinal cohorts are warranted to clarify the causal relationship between MGD and KC, to evaluate the impact of MGD treatment on KC progression, and to explore the underlying molecular and biomechanical mechanisms linking these two conditions.

## Figures and Tables

**Table 1:** Demographic Data (Number of Participants, %Females, Mean Age ± SD, Age Range, Median Age (Q1, Q3) of all Participants and of the keratoconus and control participants.

		All Participants	Keratoconus	Control	*p*
	*N*	44	17	27	0.68
		(60 eyes)	(33 eyes)	(27 eyes)	
	Female	19 (43.2%)	8 (47.1%)	11 (40.7%)	
Age	Mean ± SD	32.2 ± 10.5	29.2 ± 7.7	34.2 ± 11.7	0.25
	Range	18–56	19–50	18–56	
	Median	29.0	27.0	32.0	
	(Q1, Q3)	(24.3, 36.5)	(24.5, 31.5)	(24.0, 48.0)	

## Data Availability

The original contributions presented in this study are included in the article. Further inquiries can be directed to the corresponding author(s).

## Abbreviations

The following abbreviations are used in this manuscript:

MGD	Meibomian gland dysfunction
KC	keratoconus
PA	Palestinian Authority
VA	visual acuity
TBUT	tear breakup time
MG	meibomian gland
MQS	meibomian quality score
MES	meibomian expressibility score
OSDI	Ocular Surface Disease Index
